# The Antiseptic Treatment of Typhoid Fever

**Published:** 1882-11

**Authors:** 


### THE ANTISEPTIC TREATMENT OF TYPHOID FEVER.

At a meeting of the Société Médicale-des Hôpitaux, June 9th, M. Ferrand presented the candidate's thesis of Prof. Desplat of Lillie, upon the comparative action of carbolic acid and salicylate of soda. The views presented were that the above drugs were excellent antipyretic and antialgesic agents—sure, rapid, and permanent in their action, but, at the same time, easily eliminated, and, therefore, but slightly dangerous. Except in acute rheumatism, M. Desplat did not find any marked difference in their action. The discussion which ensued turned upon the use of carbolic acid in typhoid fever. Thirteen members took part and related their experience. Three or four did not commit themselves ; the remainder agreed in saying that the drug in question, used as recommended, had a dangerous tendency to depress the system, and to produce pulmonary congestion, exhausting sweats, and albuminuria or polyuria. It was unanimously voted that the use of carbolic acid in typhoid fever, when given as recommended (in half-gramme or gramme doses by enema twice a day), was dangerous, and without effect upon the course of the fever.

Dr. Ramonet, Physician-in-Chief at the Military Hospital of Boghar, in Algeria, has contributed an article upon the use of carbolic acid in typhoid fever, expressing directly contrary views to the above He is a follower of Desplat, except that he uses smaller doses, generally not more than two grammes per diem, by injection. The effect, he says, is to lower the temperature nearly 4°F., and to produce a most favorable change in the progress and symptoms. He has treated forty-one cases, with a morality of only two, or 4.0 per cent. The average mortality frym this disease in the army is 21 per cent.

On August 22d, at the Academy of Medicine, M. Vulpian read a paper upon the use of salicylic acid in typhoid fever. M. Vulpian based his therapeutics upon the theory that there is a *bacillus* of enteric fever in the intestine, and that this *bacillus* ought to be ferreted out and killed with an antizymotic. Having tried iodoform, boric acid, phenate of soda, and salicylate of bismuth with no effect, he finally settled upon salicylic acid. This in daily doses of two or three grammes was ineffective, but in doses of six or seven grammes daily (gr. xl. to gr. l. every two hours!) most satisfactory results were obtained in a lowering of the fever and a general amelioration of the symptoms. M. Vulpain concluded that this drug, without being curative, had an undoubted modifying influence upon typhoid fever. He thought also that salicylic acid taken by the mouth might act as a prophylactic. The discussion which followed brought out very little. It was only evident that M. Vulpain's views were theoretical, and that the clinical tests of his reputed remedy were not at all conclusive. Salicylic acid has been tried in Germany and America with no very good results results, as yet reported.—*Canada Med. and Surg. Jour.*

